# Supplementary material for: Identifying metaphors towards physiotherapists
Source: PLoS One. 2024 Dec 26;19(12):e0311304. doi: 10.1371/journal.pone.0311304 (PMC11670940; doi:10.1371/journal.pone.0311304)
Supplement: Attachment — Submitted filename: PONE-D-23-19512_Review.pdf [file pone.0311304.s001.pdf]

PLOS ONE

A QUALITATIVE STUDY ON IDENTIFYING METAPHORS TOWARDS PHYSIOTHERAPISTS

--Manuscript Draft--

**Manuscript Number:**	PONE-D-23-19512
**Article Type:**	Research Article
**Full Title:**	A QUALITATIVE STUDY ON IDENTIFYING METAPHORS TOWARDS PHYSIOTHERAPISTS
**Short Title:**	A QUALITATIVE STUDY ON IDENTIFYING METAPHORS TOWARDS PHYSIOTHERAPISTS
**Corresponding Author:**	Rabia Tugba Kilic<br>Ankara Yildirim Beyazit Universitesi<br>Çubuk/ANKARA, --- Select One --- TURKEY
**Keywords:**	Physiotherapist; Perception; qualitative method; metaphor; health education; job satisfaction
**Abstract:**	Bu çalışmanın amacı, fizyoterapistlerin fizyoterapist kavramına ilişkin sahip oldukları zihinsel imgeleri metaforlar aracılığıyla tespit etmektir. Veriler 207 fizyoterapist veya fizyoterapi öğrencisinden toplanmıştır. Her bir katılımcının “ Fizyoterapist… gibidir çünkü… ” cümlesini tamamlayan ifadelerinden oluşan ham veriler üzerinde nitel bir yöntem olan içerik analizi yapılmıştır . Fizyoterapistler için metaforlar 12 kavram etrafında toplanmıştır. Öğrenci fizyoterapistler tarafından en çok atıf yapılan metafor “ şifacı ” (%29,4) ve hizmet içi fizyoterapistler için “ yol gösterici ” (%17,2) metaforudur . " Olumsuz çağrışım " oranıHizmet içi fizyoterapistlerde " kategorisi (%7,1) öğrenci fizyoterapistlere göre %2 daha yüksekti. Kadınlar en çok " şifacı " (%21,9) kategorisini, erkekler ise en çok " şifacı " ve " destekçi " (%17,8) kategorisini vurguladı. 25 yaş altı ve 26-35 yaş grubu katılımcılar en çok " şifacı " kategorisinde yer alırken (sırasıyla %26,4 ve %18,8), 36 yaş ve üzeri katılımcılar ise en çok " yol gösteren " ve " destek veren " kategorisini tercih etmiştir." (%20,0). Fizyoterapiye meslek olarak bakış açısından hizmet içi fizyoterapist adayları ile hizmet içi fizyoterapistler arasında temel farklılıklar olduğu ve bu farklılıkların cinsiyete ve özellikle yaşa göre farklılaştığı görülmektedir. öğrencilerin çok yönlü eğitim programları ile gerçekçi bir bakış açısına sahip olmalarıdır.
**Order of Authors:**	Rabia Tugba Kilic
	Hayri Baran Yosmaoglu
**Additional Information:**	
**Question**	**Response**
**Financial Disclosure**<br><br>Enter a financial disclosure statement that describes the sources of funding for the work included in this submission. Review the submission guidelines for detailed requirements. View published research articles from *PLOS ONE* for specific examples.<br><br>This statement is required for submission and **will appear in the published article** if the submission is accepted. Please make sure it is accurate.	The author(s) received no specific funding for this work.

Powered by Editorial Manager® and ProduXion Manager® from Aries Systems Corporation

**Unfunded studies**<br>Enter: *The author(s) received no specific funding for this work.*<br><br>**Funded studies**<br>Enter a statement with the following details:<br>• Initials of the authors who received each award<br>• Grant numbers awarded to each author<br>• The full name of each funder<br>• URL of each funder website<br>• Did the sponsors or funders play any role in the study design, data collection and analysis, decision to publish, or preparation of the manuscript?<br>• **NO** - Include this sentence at the end of your statement: *The funders had no role in study design, data collection and analysis, decision to publish, or preparation of the manuscript.*<br>• **YES** - Specify the role(s) played.<br><br>* typeset	
**Competing Interests**<br><br>Use the instructions below to enter a competing interest statement for this submission. On behalf of all authors, disclose any competing interests that could be perceived to bias this work—acknowledging all financial support and any other relevant financial or non-financial competing interests.<br><br>This statement is required for submission and **will appear in the published article** if the submission is accepted. Please make sure it is accurate and that any funding sources listed in your Funding Information later in the submission form are also declared in your Financial Disclosure statement.<br><br>View published research articles from *PLOS ONE* for specific examples.	The authors have declared that no competing interests exist.

Powered by Editorial Manager® and ProduXion Manager® from Aries Systems Corporation

**NO authors have competing interests**<br><br>Enter: *The authors have declared that no competing interests exist.*<br><br>**Authors with competing interests**<br><br>Enter competing interest details beginning with this statement:<br><br>*I have read the journal's policy and the authors of this manuscript have the following competing interests: [insert competing interests here]*<br><br>* typeset	
**Ethics Statement**<br><br>Enter an ethics statement for this submission. This statement is required if the study involved:<br><br>• Human participants<br>• Human specimens or tissue<br>• Vertebrate animals or cephalopods<br>• Vertebrate embryos or tissues<br>• Field research<br><br>Write "N/A" if the submission does not require an ethics statement.<br><br>General guidance is provided below. Consult the submission guidelines for detailed instructions. **Make sure that all information entered here is included in the Methods section of the manuscript.**	Ethical approval of the study was obtained from Ankara Yildirim Beyazit University Ethics Committee (Date: 15.03.2019, #119).

Powered by Editorial Manager® and ProduXion Manager® from Aries Systems Corporation

**Format for specific study types**<br><br>**Human Subject Research (involving human participants and/or tissue)**<br>• Give the name of the institutional review board or ethics committee that approved the study<br>• Include the approval number and/or a statement indicating approval of this research<br>• Indicate the form of consent obtained (written/oral) or the reason that consent was not obtained (e.g. the data were analyzed anonymously)<br><br>**Animal Research (involving vertebrate animals, embryos or tissues)**<br>• Provide the name of the Institutional Animal Care and Use Committee (IACUC) or other relevant ethics board that reviewed the study protocol, and indicate whether they approved this research or granted a formal waiver of ethical approval<br>• Include an approval number if one was obtained<br>• If the study involved *non-human primates*, add *additional details* about animal welfare and steps taken to ameliorate suffering<br>• If anesthesia, euthanasia, or any kind of animal sacrifice is part of the study, include briefly which substances and/or methods were applied<br><br>**Field Research**<br><br>Include the following details if this study involves the collection of plant, animal, or other materials from a natural setting:<br>• Field permit number<br>• Name of the institution or relevant body that granted permission	
**Data Availability**<br><br>Authors are required to make all data underlying the findings described fully available, without restriction, and from the time of publication. PLOS allows rare exceptions to address legal and ethical concerns. See the PLOS Data Policy and FAQ for detailed information.	No - some restrictions will apply

A Data Availability Statement describing where the data can be found is required at submission. Your answers to this question constitute the Data Availability Statement and **will be published in the article**, if accepted.<br><br>**Important:** Stating 'data available on request from the author' is not sufficient. If your data are only available upon request, select 'No' for the first question and explain your exceptional situation in the text box.<br><br>Do the authors confirm that all data underlying the findings described in their manuscript are fully available without restriction?	
**Describe where the data may be found in full sentences. If you are copying our sample text, replace any instances of XXX with the appropriate details.**<br><br>• If the data are **held or will be held in a public repository**, include URLs, accession numbers or DOIs. If this information will only be available after acceptance, indicate this by ticking the box below. For example: *All XXX files are available from the XXX database (accession number(s) XXX, XXX.).*<br>• If the data are all contained **within the manuscript and/or Supporting Information files**, enter the following: *All relevant data are within the manuscript and its Supporting Information files.*<br>• If neither of these applies but you are able to provide **details of access elsewhere**, with or without limitations, please do so. For example:<br><br>*Data cannot be shared publicly because of [XXX]. Data are available from the XXX Institutional Data Access / Ethics Committee (contact via XXX) for researchers who meet the criteria for access to confidential data.*<br><br>*The data underlying the results presented in the study are available from (include the name of the third party*	Analyzed version available

and contact information or URL).<br>• This text is appropriate if the data are owned by a third party and authors do not have permission to share the data.<br><br>* typeset	
Additional data availability information:	

Manuscript

Click here to access/download;Manuscript;Manuscript Metafor.docx

A QUALITATIVE STUDY ON IDENTIFYING METAPHORS TOWARDS PHYSIOTHERAPISTS

Rabia Tugba KILIC [1], Hayri Baran YOSMAOGLU [2]

[1] **Rabia Tugba KILIC, PhD, Assist. Prof.,** Faculty of Health Sciences, Department of Physiotherapy and Rehabilitation, Ankara Yıldirim Beyazit University, TURKEY.

https://orcid.org/0000-0002-3276-5097

[2] **Hayri Baran YOSMAOGLU, PhD, Prof.,** Faculty of Health Sciences, Department of Physiotherapy and Rehabilitation, Baskent University, TURKEY.

https://orcid.org/0000-0001-7356-8500

Corresponding Author: Rabia Tugba KILIC

Post adress: Ankara Yildirim Beyazıt University**,** Esenboğa Campus, Dumlupınar Neighborhood, ANKARA /TURKEY.

Tel: +90 312 906 19 08

Fax: +90 312 906 29 67

e-mail: rtugbakilic@gmail.com

Declarations

Funding : No funding was received for conducting this study.

Disclosure statement: The authors report there are no competing interests to declare.

Authors' contribution : All authors passed four criteria for authorship contribution based on recommendations of the International Committee of Medical Journal Editors. All authors contributed to the study conception and design. Material preparation, data collection and analysis were performed by Rabia Tugba KILIC and Hayri Baran YOSMAOGLU. The first

draft of the manuscript was written by Rabia Tugba KILIC and all authors commented on previous versions of the manuscript. All authors read and approved the final manuscript.

Compliance with Ethical Standards

Ethics approval : This study was performed in line with the principles of the Declaration of Helsinki. Approval was granted by the Ethics Committee of University X (Date: 15.03.2019, #119).

Consent to participate: Informed consent was obtained from all individual participants included in the study.

Disclosure of potential conflicts of interest : The authors declare no conflicts of interest and do not have any financial disclosures.

Acknowledging statement: If our article is accepted for publication, we declare that we agree to perform a copyright transfer to your journal.

ABSTRACT

The aim of this study was to identify mental images that physiotherapists have regarding the concept of physiotherapist through metaphors. Data were collected from 207 physiotherapists or physiotherapy students. Content analysis, a qualitative method, was performed on the raw data, which was composed of each participant's statement completing the following sentence "*Physiotherapist is like… because…*". Metaphors for physiotherapists were centered around 12 concepts. Most cited metaphor by student physiotherapists was "*healer*" (29.4%) and it was "*guiding*" (17.2%) for in-service physiotherapists. The rate of the "*negative connotation*" category in in-service physiotherapists (7.1%) was 2% higher than that of the student physiotherapists. Women mostly emphasized the "*healer*" (21.9%) category, while men mostly emphasized the "*healer*" and "*supporter*" (17.8%) categories. Participants aged under 25 and

26-35 age group were mostly in the "*healer*" category (26.4% and 18.8%, respectively) but the participants aged 36 years and over mostly opted for "*guiding*" and "*supporter*" (20.0%). There were fundamental differences between student physiotherapists and in-service physiotherapists in terms viewpoint regarding physiotherapy as a profession, and that these differences vary by gender and especially by age. During the undergraduate period, it will be beneficial to ensure that students have a realistic perspective with versatile training programs.

Keywords: Physiotherapist, perception, qualitative method, metaphor, health education, job satisfaction.

INTRODUCTION

In the word metaphor, whose origin is based on the Greek word "metapherein", "meta" means “change” and "pherein" means “transfer”. [1] Metaphor was defined as a concept, fact, or event by analogy with another concept, fact, or event. [2-5] For this reason, metaphors appear in learning difficult-to-understand abstract concepts via analogies with known concepts. [6] Metaphors act as a bridge between newly learned knowledge and the existing knowledge. [7]

The main functions of metaphors can be listed as follows: creating a mental model; emphasizing the similarities and differences; a blueprint and archetype of professional thought; and a powerful mental mapping and modeling mechanism for individuals to understand and construct their own world; and, they are used as an important tool in education and training. [8-16]

According to report published by the World Physiotherapy Confederation, the duties and responsibilities of physiotherapists can be summarized as follows; to make clinical decisions,

developing a treatment plan, consulting with other healthcare professionals, implementing the treatment program, and, advising patients for self-regulation. [17, 18]

Metaphor studies is very important in educational sciences. They have been conducted to reveal the ways of the perception of different concepts in different fields. [15, 19-28] There are publications on the perception of medicine and nursing. [24, 29-31] However, to the best of our knowledge, regarding people's perceptions of the physiotherapist identity via metaphors, there exists no study in the literature.

From this point of view, it was aimed to identify the mental images that physiotherapists have about the concept of physiotherapist through metaphors, considering that it will contribute to the trainings, regulations, and studies to improve the profession of physiotherapy.

METHODS

Research Model

In this study, it was aimed to identify in-service and student physiotherapists' perceptions of physiotherapist with the help of metaphors. For this purpose, a phenomenological research design was used within the framework of the qualitative research tradition. The phenomenological pattern deals with the phenomena that are recognized but cannot be understood in depth and detail. Phenomena can occur in various forms such as events, experiences, perceptions, orientations, concepts, and situations [32].

Ethical approval of the study was obtained from X University Ethics Committee (Date: 15.03.2019, #119).

The questionnaires for this study were administered from April 15, 2019, to November 20, 2019.

Study Group

The study group of the research consists of in-service and student physiotherapists. Convenience sampling, one of the non-random sampling methods, was used in determining the physiotherapists participating in the study. In this sampling, the sampling starts with the easily accessible respondents until the researcher reaches the required sample size. [33, 34] In this direction, the metaphors of 207 in-service and student physiotherapists were examined. Then, 57 metaphors that were determined to be weak were eliminated and 150 metaphors were analyzed. Within the scope of the study, "I" abbreviation was used for in-service physiotherapists, and "S" abbreviation was used for student physiotherapists. In addition, each participant was given a number and represented by codes such as "S5" and "I2".

Data Collection Tool

In the study, in order to identify the mental images about the concept of "*physiotherapist*", the participants were asked to complete this statement: "*Physiotherapist is like... because ...*". The metaphor image specified with the concept of "*like*" in the first part of this expression pattern points out the link between the subject of the metaphor used and the source of the metaphor. In the "*because...*" part of the expression pattern, the participants provide a justification or a logical basis for this connection they have established in their own metaphors. An online questionnaire was prepared to identify the metaphors of the participants (metaphorical expression patterns) and delivered to in-service and student physiotherapists. These documents that were collected through voluntary participation constitute the main data source of the research.

Data Analysis

The data obtained in the research were analyzed by content analysis. [32] The metaphors revealed by the participants and the conceptual categories created for these metaphors are

presented in relation to the literature. To analyze the metaphors developed by the participants: a process consisting of five stages was followed: (1) coding and sorting, (2) compiling a sample metaphor image, (3) developing category, (4) establishing validity and reliability, [35] and (5) quantitative data analysis.

Coding and Sorting: In the present study, 207 raw data were reached in total. The metaphor images created by the participants were arranged in an alphabetical order and a temporary list was created using these raw data. In the data collected, whether a certain metaphor was defined clearly was checked and the metaphors presented by each participant were coded (medicine, hope, mother, magician, etc.). At this stage, the answer sheets that did not specify any metaphor images, or those with the personal thoughts of the participants in general, instead of about metaphor images, were removed from the data set. Similarly, although some participants used a metaphor image, it was found that they left the "*because* ..." part blank and did not present any logical basis for the metaphor in question. Based on these reasons, 57 metaphors that were determined to be weak were excluded from data analysis.

Compilation of Sample Metaphor Images: A total of 150 valid metaphors were found in the present study, and 88 metaphor images in these metaphors were identified and listed. After the extraction process, the metaphor documents were revised and a "*sample metaphor statement*" considered to be representing each metaphor well was chosen. While it was seen that a common justification was expressed in some metaphors, in some other metaphor images, the justification after the "*because...*" part differed. For example, using the metaphor image of "mother" for the physiotherapist phenomenon, I14 uses the love aspect of the mother image as a logical basis with the expression "*unconditionally loves patients like mothers do*", and I12 highlights the "*healer*" image of mother by stating "*mothers try to heal their children*". Furthermore, more than one "*sample metaphor statement*" was created for some metaphors in which different logical reasons were proposed. Thus, a "*sample metaphor list*" was prepared for each of the 88

metaphor images along with the compilation of metaphor patterns that were supposed to represent them best. One of the reasons for the creation of this list was that there was a need for a source consisting of representative expressions to be used in the process of collecting metaphor images under a certain category. Another reason was to increase the validity of the data analysis and interpretation process of the research.

Category Development: At this stage, the metaphor images produced by the participants were examined in terms of their common features regarding the physiotherapist phenomenon. In this process, how each metaphor image conceptualizes the phenomenon of physiotherapist was examined by considering the "*sample metaphor list*". To this end, each metaphor created by the participants was analyzed in terms of the subject of the metaphor, and the source of the metaphor. [35] Then, a total of 12 different conceptual categories were created by associating each metaphor image with a certain theme with its perspective of the physiotherapist. From this point of view, the metaphors used by the participants were grouped under 12 conceptual categories: "*guiding*", "*friendly*", "*patient/resilient*", "*who is needed*", *"surreal", "supporter/helping", "negative connotation", "healer", "versatile thinker", "someone connecting to life/giving hope", "someone adding meaning to life"*, and *"someone working consistently and regularly".*

Establishing Validity and Reliability[illegible]alidity and reliability are two very important criteria in terms of ensuring the credibility of the research results. One of the important criteria to ensure validity in a qualitative study is to report in detail the data and to explain how the results of the study were achieved. [32]. To this end, the steps followed in the data analysis process were explained in detail in order to ensure the validity of the results of the research. In addition, for each of the 88 different metaphor images obtained in the study, sample metaphor patterns that were considered to represent them best were compiled and these metaphor patterns were included in the findings section. In order to ensure the reliability of the study, an expert on

educational sciences who had previously conducted research on metaphor was consulted. This expert was asked to classify the metaphors in most appropriate 12 conceptual categories. Then, the classifications made by the expert and the researcher were compared. At this stage, Miles and Huberman's (1994) formula (Reliability = consensus / consensus + disagreement) was used for reliability calculation. In order to ensure reliability in qualitative studies, the agreement between expert and researcher evaluations for the categories is expected to be at least 80%. [36, 37] As a result of the calculations made in this research, it was found that there was a 90% agreement.

Quantitative Data Analysis: In this study, after the development of 12 conceptual categories, all data were transferred to the SPSS 24 statistical program. Then, the number (n) and percentage (%) of the participants representing 150 metaphors and 12 conceptual categories were calculated. In addition, the Chi-Square (2) test was conducted in variables such as gender, age, and the status of being an in-service physiotherapist or student physiotherapist.

RESULTS

Of the participants, 105 were female (70%), 45 (30%) were male, 51 were students (34%), and 99 (66%) were in-service physiotherapists. The metaphor images produced by the in-service and student physiotherapists about the concept of "*physiotherapist*" and the findings regarding the conceptual categories

Findings on conceptual categories

There was a total of 150 valid metaphors related to the concept of physiotherapist. In these metaphors, 88 different metaphor images were used, and these metaphor images were collected under 12 different conceptual categories as follows (Table 1).

Physiotherapist as a guide

This category involves 20 (13.3%) in-service and student physiotherapists and 14 metaphor images. The guide (f=3), pathfinder (f=4), and mother (f=2) metaphor images occurred more frequently, and other metaphor images were mentioned once. One of the participants, I163, stated for the metaphor image of the “*guide*” that “*the physiotherapist educates and guides patients regarding which way to go regarding their complaints*”. For the "*guiding*" metaphor image in this category, I156 said: "*In many cases, the physiotherapist shows the way and methods of getting health to the person who consults him/her."* Regarding the metaphor image of the mother, the following statement was said: “*the physiotherapist teaches everything again*” (S118).

Physiotherapist as a friend

Regarding the physiotherapist as a friend category, the followings were considered: friendship, closeness, and kinship relations. The sincerity and reliability of aspects of the concept of friendship were highlighted. In this context, the *"wild card/joker"* (a person who is good at everything) metaphor image was explained as follows: “… *The physiotherapist is like a son, a sibling, or sometimes a confidant*” (I156). The metaphor image of “*family member*” was expressed as follows: “*Most of the time, the physiotherapist looks after the patient more than the family; he is a brother or sister who constantly thinks about how to improve the patient even at night*” (S151).

Physiotherapist as someone patient/resilient

In this category, there are three metaphor images: ‘*patient as stone/very patient*’ (f=1)’, ‘*diamond (f=1)*’ and ‘*rock (f=1)*’. One of the participants, I161, explained his/her perspective towards the physiotherapist with the metaphor image of 'rock' as follows: “*the physiotherapist’s work area is tiring, his colleagues are challenging, working conditions are hard. Therefore, she/he must be like a rock in order to survive.*" For the “*diamond*” metaphor image, a participant stated: “*at least as durable as diamond*” (I19).

Physiotherapist as someone needed

The physiotherapist as someone needed category includes 8 (5.3%) participants and 8 different metaphor images. While a participant expressed the physiotherapist in a "*healer*" metaphor image as "*Everyone needs a physiotherapist*" (S181), another participant explained the "*water*" metaphor image as "You need it at every moment of your life. " (I184)

Physiotherapist as a surreal being

In the physiotherapist as a surreal being category, for the "*magician*" metaphor image, the following justification was given by S108: "*The moment he touches, he returns everything to normal. It gives happiness not only to the sick but to everyone."* For *"dreams full of hope*", which is another metaphor image in this category, this was said: "*Because the physiotherapist does his/her best for you to perform the functions that you thought you could never do*" (S85).

Physiotherapist as supporting/helping

In this category, "*life coach*" (f=2) metaphor image was the leading one. The participants explained the "*life coach*" metaphor image as "*the physiotherapist should be able to support the patient from every angle*" (S64) and "*because the physiotherapist helps the patient and the individual in all kinds of matters*" (S117). For the metaphor image of "*supporter*" in this category, the following expression was used: "*a supporter who can understand best both the patient and the patient's caregivers*" (S87).

Physiotherapist as negative connotation

In the negative connotation conceptual category, the metaphor image of "*donkey*" (f=2) was repeated. The following expressions were used for this metaphor image: "*because the physiotherapist works and works, does not get a proper salary, and does not get the value he/she deserves*" (I139) and "*no matter how hard the physiotherapist works, he/she cannot gain much*" (I140). The metaphor image of the "*machine*" expressed as negative opinion about the

physiotherapist as follows: "*because it is the most oppressed occupational group among the gears of the sector*" (I106).

<u>*Physiotherapist as healer*</u>

The conceptual category of physiotherapist as healer constitutes 20.6% (f=31) of the participants. The most frequently recurring metaphor images in this category are 'medicine' (f=4) and "*healing*" (f=4). These expressions were used for the metaphor image of "*medicine*": "*the physiotherapist approaches the injury like a medicine given by the doctor and heals the injury*" (I182), "*the physiotherapist is a remedy for troubles*" (S207), "*the physiotherapist heals the patients* (S147) ", and "*the physiotherapist heals*" (I189). The metaphor image of "*healing*" in this category was explained as: "*the physiotherapist is a means of healing using only his/her hands*" (I89); and "*the physiotherapist helps improve or maintain our health*" (S37). In addition to these, the other metaphor images that were frequently repeated were "*doctor*" (f=3) and "*mother*" (f=3). The following expressions were used for the metaphor image of "*doctor*": "*the physiotherapist is the person authorized to distribute healing*" (I100) and "*the physiotherapist heals the patient*" (S59). These expressions were used for the "*mother*" metaphor image: "*mothers try to heal their children*" (I199) and "*the physiotherapist always wants the good of people and tries to heal them*" (S38).

<u>*Physiotherapist as versatile thinker*</u>

The metaphor image that stands out in the versatile thinker conceptual category is the analogy of "*Swedish army knife*" (f = 2). This expression was used for the "*Swedish army knife*" metaphor image: "*the physiotherapist can approach different problems differently; s/he has a wide range of solutions and seeks to solve problems in multiple ways; s/he has a broad perspective*" (S71). The metaphor image of the "*mother*" was explained as: "*the physiotherapist takes responsibility in order to reach the physical, mental, and social well-being of the patients*" (I13).

Physiotherapist as one who connects to life and gives hope

In the conceptual category that one connects to life and gives hope, the metaphor image of "*hope*" (f=5) was the most frequently repeated metaphor. The following expressions were used regarding the metaphor image of "*hope*": "*while thinking that everything is over, everything starts again thanks to the physiotherapist*" and "*it is an orthopedic patient's hopes that the physiotherapist ends his/her pain*" (S116). S97 made the following explanation for the "*life-saving*" metaphor image in this category: "*the physiotherapist is a blessing given to people who despair of life*." The metaphor image of the "*sun*" was explained as follows: "*the physiotherapist, s/he is illumination of the darkness in the patient's world with his/her light, and s/he will be a ray of hope for the patient. S/he warms and enlightens the world of those who are cold with illness*" (I132).

Physiotherapist who adds meaning to life

In the conceptual category of one adds meaning to life, the metaphor image of "*life*" (f=3) was repeated frequently. I157 expressed the metaphor of "*life*" as follows: "*because the physiotherapist adds meaning to life*.", and I95 expressed as follows: "*the physiotherapist makes life livable.*" The following expression was used for the metaphor image of "*mother's hand*": "*physiotherapist provides a beautiful and meaningful to life with his/her treatment*" (I197). The "*cream*" metaphor image was explained as follows: "*without the physiotherapist, the patient can continue his/her life, but there is no comfort in life*" (I122).

Physiotherapist as someone working consistently and regularly

The metaphor images of "*mother*" (f=2) and "*comrade*" (f=2) were repeated in the conceptual category of someone working consistently and regularly. The following expression was used for the metaphor image of "*mother*": "*the physiotherapist treats his/her patient best for a long time*" (S11). For the "*comrade*" metaphor image, the following expression was used: "*The*

physiotherapist walks a long way with the patients in order to bring them to where they want to be" (S130). The "*clock*" image in this category was explained as follows: "*because the physiotherapist treats on an hourly basis. They work regularly*" (S70).

Conceptual categories and findings regarding sex, age, and the status of being an in-service or student physiotherapist

Chi-square analysis results regarding the relationships between conceptual categories and participants' sex, age, and the status of being an in-service or student physiotherapist are presented in Table 2.

Table 2 shows that the student physiotherapists proposed metaphors mostly in the "*healer*" (29.4%) category, which was followed by the metaphors in the categories of "*someone connecting to life/giving hope*" (17.6%) and "*supporter-helping*" (13.7%), in this order. In addition, student physiotherapists proposed very few metaphors in the "*negative connotation*" (2.0%) category. An analysis of the distribution of metaphors proposed by in-service physiotherapists by the conceptual categories showed that, unlike student physiotherapists, more metaphors were created in the conceptual category of "*guiding*" (17.2%), which was followed by "*healer*" (16.2%) and "*supporter/helping*" (12.1%), in this order; and, in-service physiotherapists proposed more "*negative connotation*" metaphors (7.1%) than student physiotherapists did (2.0%).

An analysis of the distribution of the conceptual categories by gender showed that women mostly proposed metaphors in the category of "*healer*" (21.9%), which was followed by "*guiding*" (16.2%) and "*someone connecting to life/giving hope*" (12.4%). The female participants placed the least emphasis on the "*patient/resilient*" (1.9%) aspect of the physiotherapist. An analysis of the metaphors of the male participants showed that the categories of "*healer*" (17.8%) and "*supporter/helping*" (17.8%) were equally emphasized.

Furthermore, it was observed that the categories that demonstrate the characteristics of the physiotherapist as "*someone connecting to life/giving hope*" (11.1%) and "*versatile thinker*" (11.1%) were both heavily emphasized. Although the “*guiding*” category was prominent in the metaphors of the female participants, it was found that the rate of male participants proposing in this category was quite low (6.7%). Moreover, unlike women, the male participants did not propose any metaphors in the "*surreal*" category.

The distribution of conceptual categories by age variable was also examined in the study. Participants aged 25 years and under mostly proposed a metaphor in the "*healer*" (26.4%) category. Moreover, the metaphors in the categories of "*connecting to life/giving hope*" (16.7%) and "*supporter/helping*" (11.1%) were also mostly in this age group. Similarly, the category of "*healer*" (18.8%) had a great number of the metaphors form the 26-35 age group. However, it was observed that the rate of metaphors emphasizing the “*guiding*” feature of the physiotherapist (14.6%) was higher in the 26-35 age group than the participants aged 25 years and less. Participants aged 36 years and over proposed metaphors mostly in the categories of "*guiding*" (20.0%) and "*supporter/helping*" (20.0%). Although it was not possible to make interpretation regarding the statistical significance of the difference, it was observed that as the age increased, the rate of the participants' metaphors in the “*guiding*” category also increased, but the rate in the "healer" and "*someone connecting to life/giving hope*" categories decreased.

DISCUSSION

In the present study, in order to determine the mental images that in-service and student physiotherapists have regarding the concept of physiotherapist through metaphors. Then, a qualitativ[illegible] method of content analysis was performed on the obtained raw data. Consequently, metaphors reflecting the participants’ perspectives regarding the physiotherapist were identified in relation with their age, sex, and status of being an in-service or student physiotherapist.

The first important finding of this study was that the student physiotherapists mostly constructed metaphors in the "*healer*" category, and in-service physiotherapists, on the other hand, proposed metaphors in the conceptual category of "*guiding*". The probable reason for this outcome is that, in the courses they take throughout their education life, physiotherapy students are exposed to the knowledge that the treatments given by physiotherapists are aimed to improve a single factor; however, as they gain experience as an in-service physiotherapist, they may see that the treatments applied in the field of physiotherapy are multifactorial, and that biopsychosocial, and holistic approaches have an important place in treatment. Physiotherapists, who have gained the awareness with time and experience that the treatment should be multi-faceted, including the affected physical and emotional structures, think that their profession is important in the stages of guiding and educating patients for the recovery steps, rather than just the actual recovery; therefore, they constructed metaphors largely in the "*guiding*" conceptual category.

Another important finding of the study is that the mental images acquired regarding the concept of physiotherapist are affected by gender. While the women proposed metaphors mostly in the categories of "*healer*", "*guiding*", and "*connecting to life*", in this order; men proposed metaphors mostly in "*healer*" and "*supporter/helper*" categories equally, which were followed by the categories of "*connecting to life/giving hope*" and "*versatile*". The reason for this difference may be the effect of gender difference and reflection of the roles of the individuals in society. The concepts of sex and gender concepts can be defined biologically and socially. Basically, sex refers to the biological differences of men and women. On the other hand, the concept of being a woman or a man in daily life, which is formed by the society according to the biological sex of the person, refers to gender. In other words, while sex defines the anatomical difference between men and women; gender refers to the social construction difference between men and women. [38] There are many studies on gender roles and patterns.

Research shows that men are expected to be strong and able to support their families, and women, on the other hand, are expected to be patient, understanding, being able to look after home/family, and organizing human relations. It has been observed that there are the expectations for male and female roles in the traditional society and family system. [39, 40] Research findings on the roles of women and men from different countries present similar results. While men were attributed to features such as strong, rude, adventurous, aggressive, dominant, independent, and self-confident; and women are attributed features such as dependent, weak, polite, emotional, sensitive, talkative, caring, and helpful. [41] The gender roles and stereotypes that society expects the individual to adopt affect the way they perceive themselves [42]. We think that the difference in the imageries between men and women in the present study and the prominence of the "*supporter/helper*" metaphor as well as the "*guiding*" metaphor in male participants may be the result of male individuals being affected by gender roles.

According to the presented study findings, distribution of conceptual categories by age variable also varied. The participants aged 25 years and under, and similarly, those in the age range of 26-35 years concentrated on the “*healer*” category; however, the rate of 26-35-year-old participants proposing the “*guiding*” metaphor was higher than that of the participants aged 25 years and less. Participants aged 36 years and over proposed metaphors mostly in the categories of "guiding" and "*supporter/helping*". Based on these findings, as age increased, the rate of the participants' metaphors in the "*guiding*" category also increased. On the other hand, it was observed that their rate in the "*healer*" and "*connecting to life/giving hope*" categories decreased. Aging is as much a social category as it is a biological category. [43] Aging has been addressed by many disciplines such as medicine, psychology, sociology, and anthropology, and it has been the subject of academic studies from various vantage points. However, it is also very important to reveal the meaning of aging. At this point, philosophy plays a role. When

considered from Plato's point of view, old age is a period of wisdom. [44] Studies [45, 46] presenting aging as a maturation period and stating that people are more knowledgeable and better equipped in terms of virtue explain the increase in the high rate of the participants' metaphors in the "*guiding*" category in the present study.

To the best of our knowledge, there exists no metaphor study about the concept of physiotherapist in the literature. However, in a metaphor study related to the concept of "*physician*", it was observed that all the metaphor categories contained only positive features and positive perceptions. The physician candidates mostly associated their physician identity with the "sacrifice" category, which was followed by "*wisdom*", "*compulsory/required*", "*creativity*", and "*leadership*" categories, in this order. [47] These categories are similar to the ones found in the present study: "*guiding*", "*friendly*", "*patient/resilient*", "*who is needed", "supporter/helping", "healer", "versatile thinker", "someone connecting to life/giving hope", "someone adding meaning to life",* and *"someone working consistently and regularly".* Unlike the study about "*physicians*", the present study contained "*negative connotation*" metaphor images for the physiotherapist. It was determined that the students formed a very little metaphor for the concept of physiotherapist in the "*negative association*" category; however, it was found that this ratio was higher in in-service physiotherapists compared to student physiotherapists. To this end, working conditions after graduation, job status, the reputation of being a physiotherapist, professional regulations, and other disciplines' point of view regarding the field of physiotherapy are thought to contribute to the formation of the differences in this imagery.

Conclusion

There were fundamental differences between student physiotherapists and in-service physiotherapists in terms viewpoint regarding physiotherapy as a profession, and that these differences vary by gender and especially by age. During the undergraduate period, it will be beneficial to ensure that students have a realistic perspective with versatile training programs

improving working conditions via occupational laws and regulations may also be beneficial in order to increase high working satisfaction rates and more optimist point of view can be obtained.

Disclosure statement: The authors report there are no competing interests to declare.

REFERENCES

1. Levine, P. M. (2005). Metaphors and images of classrooms. *Kappa Delta Pi Record, 41*(4), 172-175.
2. Goldstein, L. S. (2005). Becoming a teacher as a hero's journey: Using metaphor in preservice teacher education. *Teacher Education Quarterly, 32*(1), 7-24.
3. Saban, A. & Kocbeker, B. N. (2006). An investigation of the concept of teacher among prospective teachers through metaphor analysis. *Educational Sciences: Theory & Practice, 6*(2).
4. Arnett RC. Metaphorical guidance. Journal of Educational Administration. 1999.
5. Palmquist R. Metaphors are good mirrors: Reflecting on change for teachwer educators. Journal of academic Librainship. 2001;27(1):185-9.
6. Gecit, Y., & Gencer, K. (2011). Determining The Geographical Perception Of The 1st. Grade Students In The Department Of Primary Education Through Metaphor (Example Of Rize University) *Marmara Geographical Journal Archive,* 23, 1-19
7. Arslan, M. M., & Bayrakci, M. (2006). Examination of Metaphoric Thinking and Learning Approach in Terms of Education. *National Education, 35*(171), 100-108.
8. Saban A. Prospective classroom teachers' metaphorical images of selves and comparing them to those they have of their elementary and cooperating teachers. *International Journal of Educational Development.* 2004;24(6):617-35.

9. Ben-Peretz M, Mendelson N, Kron FW. How teachers in different educational contexts view their roles. *Teaching and Teacher Education.* 2003;19(2):277-90.

10. Cook-Sather A. Movements of mind: The matrix, metaphors, and re-imagining education. *Teachers college record.* 2003;105(6):946.

11. Gillis C, Johnson CL. Metaphor as renewal: Re-imagining our professional selves. *The English Journal.* 2002;91(6):37-43.

12. Mahlios M, Maxson M. Metaphors as structures for elementary and secondary preservice teachers' thinking. *International Journal of Educational Research.* 1998;29(3):227-40.

13. Fenwick T. Adventure guides, outfitters, firestarters, and caregivers: Continuing educators' images of identity. *Canadian Journal of University Continuing Education.* 2000;26(2).

14. Hoban G. Using a reflective framework to study teaching-learning relationships. *Reflective practice.* 2000;1(2):165-82.

15. Afacan O. F. Metaphor statuses of pre-service science teachers for the concepts of "science" and "science and technology teacher". *Education Sciences.* 2011;6(1):1242-54.

16. Martínez MaA, Sauleda Ns, Huber GL. Metaphors as blueprints of thinking about teaching and learning. *Teaching and Teacher Education.* 2001;17(8):965-77.

17. Kunduracilar Z, Akbaş E, Büyükuysal Ç, BİÇKİ D, Erdem EU. Evaluation of Professional Awareness in Physiotherapy and Rehabilitation Department Students. *Journal of Higher Education & Science.* 2012;2(3).

18. WCPT W. Policy Statement: Description of Physical Therapy. World conferance of Physical therapy. 2011.

19. Sarac, H. (2019). Metaphor Analysis Study Of Middle School 8th Grade Students About Smart Board and Mobile Phone. *PAU Journal of Education.* 45(45): p. 22-37.

20. Babacan E. AGSl Students' Perceptions on the Concept of Music: Metaphor Analysis. *Journal of Education and Instructional Studies.* 2014;3(1):124-32.

21. Tortop HS. Metaphors of Pre-service Teachers about University Teachers and Metaphor as an Evaluation Tool. *Journal of Higher Education & Science*. 2013;3(2).

22. Ekici G. Determining Biology Teacher Candidates' Perceptions of the Concept of Microscope: A Metaphor Analysis Study. *Journal of Kirsehir Education Faculty.* 2016;17(1).

23. Dös I. Metaphor Perceptions of Prospective Teachers on the Concept of Inspectorship. *Gaziantep University Journal of Social Sciences.* 2010;9(3).

24. Keskin MO, Yıldız ÖÖ, Aksakal E. Metaphorical Perceptions of Medical Faculty Students Regarding the Concept of Ethics. *Ihlara Journal of Educational Research.* 4(2):300-13.

25. Gocen G. Metaphorical Perception Of Those Who Learn Turkish As A Foreign Language Towards "Grammar Of Turkish". *Başkent University Journal Of Education.* 2019;6(1):28-45.

26. Ozyıldırım M, Sarı I. Metaphorical perceptions of trainers regarding the concept of sport psychology. *International Refereed Journal of Academic Sports Health and Medical Sciences.* 2018;29:177-94.

27. Gokce AT, Bulbul T. School is a human body: A metaphor study for vocational high school students' perceptions of school. *Journal of Educational Sciences Research.* 2014;4(1):63-88.

28. Dogan Y. The Battle of Çanakkale from the Perspective of Teacher Candidates and Students in its 100th Anniversary: A Metaphor Research. Electronic Turkish Studies. 2015;10(3).

29. Ozveren H, Ozden D, Gulnar E. Nursing First Year Nursing Students' Perception of Nursing: A Metaphor Analysis. *Journal of Health Sciences.* 27(2):162-9.

30. Kucuk S., Demir K. & Uludasdemir D.(2020). Determination of the Metaphors About Nurse Perception of 7-17 Age Hospitalized Children and Adolescents. *Journal of Education and Research in Nursing.* 17(1), 40-45.

31. Adam, M. (2020). Metaphor of BPJS Kesehatan as a Sick Patient in Indonesian Media Headlines. *ELS Journal on Interdisciplinary Studies in Humanities, 3*(1), 10-21.

32. Lune, H., & Berg, B. L. (2017). Qualitative research methods for the social sciences: Pearson.

33. Buyukozturk S, Akgun OE, Demirel F, Karadeniz S, Cakmak EK. Scientific research methods. 2015.

34. Cohen, L., Manion, L., & Morrison, K. (2013). The ethics of educational and social research. In *Research methods in education* (pp. 99-128). Routledge.

35. Saban, A. & Kocbeker, B. N. (2006). An investigation of the concept of teacher among prospective teachers through metaphor analysis. *Educational Sciences: Theory & Practice, 6*(2).

36. Miles, M. B., & Huberman, A. M. (1994). *Qualitative data analysis: An expanded sourcebook*: Sage.

37. Patton, M. Q. (2014). Qualitative Research & Evaluation Methods: Integrating Theory and Practice. *SAGE Publications.4.*

38. Cengiz, A. (2013). Women Mental Health and Gender; Application in a Clinic in Antalya, *Women's Studies and Gender Master's Thesis.*

39. Imamoglu O. Changing family roles in a changing world. *Journal of Women's Studies;* Number 1 (1993). 2012.

40. Powell, G. N., & Greenhaus, J. H. (2010). Sex, gender, and decisions at the family - work interface. *Journal of management, 36*(4), 1011-1039.

41. Best, D. L., & Williams, J. E. (1993). A cross-cultural viewpoint. In A. E. Beall & R. J. Sternberg (Eds.), *The psychology of gender* (pp. 215–248). Guilford Press.

42. Citak, A. (2008). Çıtak, Aylin (2008). "Attitudes Towards Women's Work: A Comparison by Gender, Gender Role and Socio-Economic Level". *Master Thesis. Ankara: Ankara University Institute of Social Sciences, Department of Psychology (Social Psychology).*

43. Nazli, A. (2016). Aging, individual and society: A sociological view on aging. *Ege Journal of Medicine. 55 (Supplement): 1-5*

44. Bektas, O. E. (2017). Being Elderly in a Postmodern World. *Elderly Issues Research Journal, 10*(2), 9-18.

45. Cicero, M. T. (2015). Cicero's Essays on Old Age and Friendship, Also His Paradoxes, *Creative Media Partners, LLC.*

46. P Parkin, T. G. (2003). Ageing in antiquity: status and participation. Old Age from Antiquity to Postmodernity.(ed.) P. Johnson & P. Thane. In: New York: Routledge Press.

47. Abay, S. E., Sezer, B., & Basusta, N. B. (2017). The Perceptions towards Physician's Identity among Physician Candidates: An Example of Metaphor Analysis from the Research at Hacettepe University Medical School *Journal of Continuing Medical Education, 26*(1), 1-8.

Tables

Table 1. Distribution of metaphors by categories.

Categories	*n* (%)	Metaphor Image	*f*
1. Guiding	20 (13.3)	Guide (3), Pathfinder (4), Sun (1), Light source (1), Marathon runner (1), Teacher (1), Sculptor (1), Mother (2), Light (1), Life coach (1), Street lamp (1), Advisor (1), Star (1), Operator (1).	14
2. Friend	9 (6.0)	Psychologist (1), Friend (1), Mother (2), Joker (1), Family member (1), Parent (1), Friend (1), Sister (1).	8
3. Patient/resilient	3 (2.0)	Patience (1), Rock (1), Diamond (1)	3
4. Someone needed	8 (5.3)	Smartphone (1), Mortar (1), Horizon line (1), Hero (1), Our feet and hands (1), Healer (1), Cane (1), Water (1).	8
5. Surreal	5 (3.3)	Magic (1), Magician (1), Hopeful dreams (1), Superman (1), Artist (1).	5
6. Supporter/helping	19 (12.6)	Right arm (1), Life coach (2), Angel (2), Crutches (1), Old plane tree (1), Mother (3), Support (1), Step (1), The most important part of the team (1), Family (1), Hand reaching out to the patient (1), Sidekick (1), Assistant (1), Psychologist (1), Empathy (1).	15
7. Negative connotation	8 (5.3)	Relative (1), Distant relative (1), Assistant health personnel (1), Scapegoat (1), Porter (1), Machine (1), Donkey (2).	7
8. Healer	31 (20.6)	Medicine (4), Healing (1), Master of function (1), Hope (1), Sea (1), Sculptor (2), Repairman (2), Repairer (1), Water (1), Building foundation (1), Superhero (1), Traffic police (1), Healing (4), Mother (3), Physiotherapist (1), Magic hand (1), Doctor (3), Light (1), Pain reliever (1)).	19
9. Versatile thinker	9 (6.0)	Guide (1), Sculptor (1), Mother (2), Swiss army knife (2), Architect (1), Physician (1), Life coach (1).	7
10. Someone connecting to life/giving hope	18 (12.0)	Wing (1), Psychologist (1), Life (1), Tailor (1), Hope (5), Hero (1), Life saver (2), Herald of spring (1), Sun (2), Tree (1), Life support (1), Life water (1).	12

11. Someone adding meaning to life	9 (6.0)	Life (3), Life coach (1), Doctor's helping hand (1), Cream (1), Water (1), Magician (1), Mother's hand (1).	7
12. Someone working consistently and regularly	11 (7.3)	Mother (2), Artist (1), Diamond (1), Iron (1), Comrade (2), Savior (1), Clock (1), Bee (1), Architect (1).	9

Table 2. Chi-square test results between conceptual categories sex, age, and the status of being an in-service or student physiotherapist

**Category**		**Student/In-Service Physiotherapist***		**Sex***		**Age***		
		**Student**	**In-Service**	**Female**	**Male**	**≤25**	**26-35**	**≥36**
1. Guiding	n	3	17	17	3	7	7	6
	(%)	(5.9)	(17.2)	(16.2)	(6.7)	(9.7)	(14.6)	(20.0)
2. Friend	n	2	7	6	3	7	2	0
	(%)	(3.9)	(7.1)	(5.7)	(6.7)	(9.7)	(4.2)	(0.0)
3. Patient/Resilient	n	0	3	2	1	0	3	0
	(%)	(0.0)	(3.0)	(1.9)	(2.2)	(0.0)	(6.3)	(0.0)
4. Someone needed	n	3	5	6	2	2	3	3
	(%)	(5.9)	(5.1)	(5.7)	(4.4)	(2.8)	(6.3)	(10.0)
5. Surreal	n	3	2	5	0	4	0	1
	(%)	(5.9)	(2.0)	(4.8)	(0.0)	(5.6)	(0.0)	(3.3)
6. Supporter/Helping	n	7	12	11	8	8	5	6
	(%)	(13.7)	(12.1)	(10.5)	(17.8)	(11.1)	(10.4)	(20.0)
	n	1	7	4	4	3	3	2

7. Negative Connotation	(%)	(2.0)	(7.1)	(3.8)	(8.9)	(4.2)	(6.3)	(6.7)
8. Healer	n	15	16	23	8	19	9	3
	(%)	(29.4)	(16.2)	(21.9)	(17.8)	(26.4)	(18.8)	(10.0)
9. Versatile thinker	n	3	6	4	6	4	3	2
	(%)	(5.9)	(6.1)	(3.8)	(11.1)	(5.6)	(6.3)	(6.7)
10. Someone connecting to life/giving hope	n	9	9	13	5	12	4	2
	(%)	(17.6)	(9.1)	(12.4)	(11.1)	(16.7)	(8.3)	(6.7)
11. Someone adding meaning to life	n	2	7	6	3	3	4	2
	(%)	(3.9)	(7.1)	(5.7)	(6.7)	(4.2)	(8.3)	(6.7)
12. Someone working consistently and regularly	n	3	8	8	3	3	5	3
	(%)	(5.9)	(8.1)	(7.6)	(6.7)	(4.2)	(10.4)	(10.0)
Total	n	51	99	105	45	72	48	30
	(%)	(100)	(100)	(100)	(100)	(100)	(100)	(100)

* The number of cells whose expected frequency falls below 5 constitutes approximately 41.7% of the total cells according to the variable of "in-service/student" (n=10) and "gender" (n=10); and constitutes approximately 72.2% of the total cells according to the variable of "age" (n=26). For this reason, meaningfulness interpretation was not made in this analysis, and only cross table was used.
