# Supplementary material for: Identifying metaphors towards physiotherapists
Source: PLoS One. 2024 Dec 26;19(12):e0311304. doi: 10.1371/journal.pone.0311304 (PMC11670940; doi:10.1371/journal.pone.0311304)
Supplement: Attachment — Submitted filename: Comment.docx [file pone.0311304.s002.docx]

**Comment**

***Abstract***

I wonder how 207 participants were used. Was there no point when theoretical saturation was achieved with some of the participants during the data collection?

In addition, it is not conventional to have the percentage of respondents to the questions. That’s quantitative! In qualitative, we are concerned about the views and experiences as they narrated by the study population.

***Introduction***

Please can clearly state the relevance of metaphors in physiotherapy practice? Please give examples also- be it in medicine or nursing; or even a hypothetical example in physiotherapy. The concept ‘metaphor’ rather seems abstract in your introduction.

***Method***

Please how did you determine that particular metaphors are week and discarded them?

***Result***

Please refer to my comments on the abstract.

***Discussion***

First and the second paragraphs of your discussion did not have any citations. And it is not conventional for a whole paragraph not to carry a single citation. Please structure the paragraphs in such a way they will have at least one citation each.

***Conclusion***

Please look at the conclusion again. It is only comparing fundamental differences between student physiotherapists and in-service physiotherapists in terms viewpoint regarding physiotherapy as a profession, which is not the primary aim of your study and which contravenes the principles of a qualitative research. Please revisit this. Second paragraph of the discussion section captures what can be used as your conclusion.
