# Supplementary material for: Identifying metaphors towards physiotherapists
Source: PLoS One. 2024 Dec 26;19(12):e0311304. doi: 10.1371/journal.pone.0311304 (PMC11670940; doi:10.1371/journal.pone.0311304)
Supplement: Attachment — Submitted filename: RESPONSE LETTER-PLOS ONE 2.docx [file pone.0311304.s003.docx]

**RESPONSE LETTER**

1. Please provide additional details regarding participant consent. In the ethics statement in the Methods and online submission information, please ensure that you have specified what type you obtained (for instance, written or verbal, and if verbal, how it was documented and witnessed).

The following sentence was added to the Research model section to clarify the issue:

*‘Participant approval was obtained through the question added to the beginning of the online submission form.’*

2. If your study included minors, state whether you obtained consent from parents or guardians. If the need for consent was waived by the ethics committee, please include this information.

The following sentence was added to the Research model section to clarify the issue:

*‘The study did not include minor participants.’*

3. If you are reporting a retrospective study of medical records or archived samples, please ensure that you have discussed whether all data were fully anonymized before you accessed them and/or whether the IRB or ethics committee waived the requirement for informed consent. If patients provided informed written consent to have data from their medical records used in research, please include this information.

We did not include such information because we did not perform a retrospective study of medical records or archived samples.
