# Supplementary material for: Identifying metaphors towards physiotherapists
Source: PLoS One. 2024 Dec 26;19(12):e0311304. doi: 10.1371/journal.pone.0311304 (PMC11670940; doi:10.1371/journal.pone.0311304)
Supplement: Attachment — Submitted filename: 2024.docx [file pone.0311304.s004.docx]

Reviewer #1:

1. Thank you for the opportunity to read the revised manuscript. My previous comments and observations were addressed. However, there is a minor issue in the present draft. The sentence in line 407-408 seems incomplete, kindly modify.

Answer: The sentence on lines 407 and 408 has been changed as follows.

*‘In the present study, the mental images that in-service and student physiotherapists have regarding the concept of physiotherapist through metaphors was determined’.*

Reviewer #3:

1. Introduction section:
   - Some references are missing in many sentences;

Answer: References were added to the sentences where references were missing.

2. Methods section:
   a. No calculation or reference was given for how the sample size was calculated;

The sampling method has already been described in the study group section of the article as follows:

*We used purposive sampling method in the sample selection of the research. Purposive sampling allows in-depth research by selecting information-rich cases depending on the purpose of the study [43]. Because of the aim of this research is to identify and compare metaphors related students and in-service physiotherapists, we included these participants in the study according to the specified criterion [44, 45]. The criterion was to be in-service or student physiotherapists and to be volunteer.*

b. No reporting statement was described or clearly reported that supported the type of study;
The mixed method of the research model is stated in the research model section of the manuscript. Additionally, it was emphasized that the qualitative part of the research was phenomenology and the quantitative part was a descriptive research. Type of the study was described in main text as follows:

*In this study, it was aimed to identify in-service and student physiotherapists’ perceptions of physiotherapist with the help of metaphors. This study is a mixed type research using qualitative and quantitative analysis methods.. This method allowed that a better understanding of a specific phenomenon by using qualitative and quantitative methods together [34]. A mixed methods research combine both qualitative and quantitative analyzes while developing research that provides more depth than only qualitative or only quantitative research can produce [35].*

3. Discussion section:
- Discussions should be reviewed in light of the overall improvement of the paper. Redundant sentences and prewritten information should be avoided. Focus on take-home messages.

Answer: The discussion has been reviewed in light of the overall development of the article. and necessary arrangements were made.
